# Improved Saliva Pump. Codman & Shurtleff's

**Published:** 1868-04

**Authors:** 


*Improved Saliva Pump. Codman & Shurtleff's,*— During Commencement Week we witnessed the application of this Saliva Pump at the Baltimore College Infirmary, and it proved so highly satisfactory that we have introduced it into our office practice. On referring to the advertising columns of the present number of the Journal, where a cut of this instrument appears, it will be seen to consist of a glass bottle or receiver, which is attached to the back of the operating chair by means of a brass frame having teeth to enter the plush of the chair, and thus hold the bottle in position. This frame once attached to the chair, need not be removed, and the plush on the back can be protected from injury by sewing on pieces of canvas or other material, into which the teeth of the frame can be inserted. From this glass bottle, which holds about half a pint, proceed two pieces of rubber tubing, one of which is attached to a bent vulcanized rubber tube for entering the mouth over the lower front teeth, with a perforated bulb on the end to allow the saliva to enter; the other piece of tubing, proceeding from the bottle alongside of the first, has attached to it a hand bellows, similar to the spray apparatus, to be worked by the patient when the lower part of the mouth becomes filled with saliva. We have found it to be a very useful instrument and well worth a trial.

